# Radiation dose and image quality of high-pitch emergency abdominal CT in obese patients using third-generation dual-source CT (DSCT)

**DOI:** 10.1038/s41598-019-52454-5

**Published:** 2019-11-04

**Authors:** Robert Forbrig, Michael Ingrisch, Robert Stahl, Katharina Stella Winter, Maximilian Reiser, Christoph G. Trumm

**Affiliations:** 1 Institute of Neuroradiology, University Hospital, LMU Munich, Munich, Germany; 2 Department of Radiology, University Hospital, LMU Munich, Munich, Germany; 3 Institute for Diagnostic and Interventional Radiology, Neuroradiology and Nuclear Medicine, München Klinik Harlaching, Munich, Germany

**Keywords:** Gastrointestinal system, Obesity, Body mass index, Computed tomography, Pain

## Abstract

In this third-generation dual-source CT (DSCT) study, we retrospectively investigated radiation dose and image quality of portal-venous high-pitch emergency CT in 60 patients (28 female, mean age 56 years) with a body mass index (BMI) ≥ 30 kg/m^2^. Patients were dichotomized in groups A (median BMI 31.5 kg/m^2^; n = 33) and B (36.8 kg/m^2^; n = 27). Volumetric CT dose index (CTDI_vol_), size-specific dose estimate (SSDE), dose length product (DLP) and effective dose (ED) were assessed. Contrast-to-noise ratio (CNR) and dose-independent figure-of-merit (FOM) CNR were calculated. Subjective image quality was assessed using a five-point scale. Mean values of CTDI_vol_, SSDE as well as normalized DLP and ED were 7.6 ± 1.8 mGy, 8.0 ± 1.8 mGy, 304 ± 74 mGy * cm and 5.2 ± 1.3 mSv for group A, and 12.6 ± 3.7 mGy, 11.0 ± 2.6 mGy, 521 ± 157 mGy * cm and 8.9 ± 2.7 mSv for group B (p < 0.001). CNR of the liver and spleen as well as each calculated FOM CNR were significantly higher in group A (p < 0.001). Subjective image quality was good in both groups. In conclusion, third-generation abdominal high-pitch emergency DSCT yields good image quality in obese patients. Radiation dose increases in patients with a BMI > 36.8 kg/m^2^.

## Introduction

Obesity, which is defined as a body mass index (BMI) of at least 30 kg/m^2^, represents a growing issue in the western societies^1^. Obese patients pose a special diagnostic challenge in the emergency setting, as the image quality of ultrasound and X-ray is limited. In this context, a better image quality is associated with a higher patient radiation dose^2^. Furthermore, both the gantry diameter and ad-hoc availability of magnetic resonance imaging are limited^3,4^. Hence, computed tomography (CT) often represents the only non-invasive and easily available examination modality to clarify the medical condition of an obese patient. As an expression of the technical adaptation to an increasingly obese patient population, modern CT scanners have larger gantry diameters of up to 80 cm, patient tables with a maximum weight capacity of 300 kg, a higher tube power simultaneously providing a stable table speed despite a higher table load, as well as a larger field of view^5^.

In this situation, dual-source CT (DSCT), which was introduced in the mid 2000s, provides simultaneous usage of two tubes^3,5,6^. However, particularly in young obese patients the optimal balance between radiation dose penalty and potential endangerment of the patient due to reduced image quality caused by dose limitation is essential.

DSCT scanners of the current, third generation have two X-ray tubes with a maximal generator power of 120 kW each, enabling tube currents up to 1300 mA each. In combination with automated dose modulation and iterative reconstruction, these features contribute to image quality and radiation dose optimization in obese patients^5,7^. Furthermore, third-generation DSCT scanners provide high-pitch protocols with a pitch factor of up to 3.2. This technique allows for the continuous scan of a complete abdominal volume in less than one second^5^, consequently reducing both motion artifacts and probably (when combined with a reduced rotation time of up to 0.25 s)^5^ radiation dose according to several studies^8–10^. In obese patients, however, these advantages in turn yield a higher image noise due to a limited tube current: CT tubes generate only a certain amount of X-radiation during the extremely short acquisition times of high-pitch CT protocols. Hence, high-pitch CT could not be applied in a non-weight-selected patient population without taking the risk of a reduced diagnostic confidence in obese patients so far. This was confirmed by high-pitch CT studies which to date mainly analyzed normal-weight individuals^10–12^.

The aim of this study was to evaluate the impact of a high-pitch CT acquisition protocol on radiation dose and image quality in obese patients, who received a portal-venous emergency CT of the abdomen on a third-generation DSCT scanner.

## Material and Methods

### Patient selection

This retrospective single-center study was approved by the responsible institutional review board (Project Number 811-16) of the Ludwig-Maximilians-University Munich with a waiver for written informed consent. The study was performed in accordance with the Declaration of Helsinki.

All examinations were carried out on a third-generation DSCT scanner (SOMATOM Force, Siemens Healthineers) between February 2015 and December 2016. Within this time span, a total of 60 patients met the following inclusion criteria:

- Age ≥ 18 years
- BMI ≥ 30 kg/m^2^
- Acute abdominal pain
- High-pitch abdominal DSCT in portal-venous phase

Non-contrast and low-dose CT, repeated CT scans of the same patient, and examinations of patients with a body weight > 300 kg were excluded from further analysis.

### CT acquisition protocol

Table 1 summarizes the CT acquisition parameters. Each patient was examined in dual-source, helical acquisition mode. Concerning the applied high-pitch dual-source mode (Flash mode, Siemens Healthineers)^13^ of third-generation DSCT, both X-ray tubes start up simultaneously, yielding substantial increase of overall tube current and thus enabling CT examinations of overweight individuals^3,5–7^. The scan range comprised the whole abdomen, extending from the diaphragm to the pubic symphysis. Automated dose modulation was enabled by default, in terms of automated tube current modulation (ATCM; CARE Dose 4D, Siemens Healthineers)^12,14,15^ and automated tube voltage selection (ATVS; CARE kV, Siemens Healthineers)^16,17^ with a quality reference tube current time product of 80 mAs and a quality reference tube voltage of 140 kV. The gantry rotation time was 0.25 seconds per rotation, the pitch 1.55, the section thickness 0.75 mm, and the slice collimation 192 × 0.6 mm. A body-weight-adapted non-ionic iodinated contrast agent (Iomeprol 400 mg iodine/ml, 1 ml per kg; Imeron, Bracco Imaging) was administered intravenously at a flow rate of 2.5 mL/s followed by 100 mL of saline. CT images were acquired using the bolus-tracking technique (CARE Bolus, Siemens Healthineers), with the region of interest (ROI) manually placed in the abdominal aorta and the trigger threshold set to 100 Hounsfield units (HU) with a 95-second delay for the portal venous phase. Advanced modeled iterative reconstruction (ADMIRE), strength 3, was enabled in each patient.

**Table 1:** CT acquisition parameters.

Scanner Acquisition mode Scan protocol	SOMATOM Force, Siemens Healthineers High-pitch, dual-source, helical Flash Abdomen
Scan area	Abdomen-Pelvis
Scan direction	Cranio-caudal
Automated dose modulation	CARE Dose 4D CARE kV (dose optimizer setting: 7)
Quality reference tube current	80 mAs
Quality reference tube voltage	140 kV
Rotation time	0.25 s
Pitch Table speed	1.55 438 mm/s
Slice width	0.75 mm
Slice collimation	192 × 0.6 mm
Reconstruction kernel	Br36
Contrast medium	Iomeprol 400 mg/mL, 1 ml per kg
Flow rate	2.5 mL/s
Start delay	95 s
Iterative reconstruction	ADMIRE, strength 3

Abbreviations: CT, computed tomography; ADMIRE, advanced modeled iterative reconstruction.

CT acquisition parameters. Abbreviations: CT, computed tomography; ADMIRE, advanced modeled iterative reconstruction.

### Radiation metrics

Individual values of selected tube voltage and tube current time product as well as the volumetric CT dose index (CTDI_vol_) and dose length product (DLP) were documented from the dose report, which was automatically stored in the picture archiving and communication system (Syngo Imaging 2010, Siemens Healthineers). The DLP was normalized for a typical abdominal scan length of 40 cm^14,15^. The normalized effective dose (ED) was then calculated by multiplying the normalized DLP with the specific conversion factor *k* for combined adult abdomen and pelvic CT of 0.017 mSv/mGy * cm^18,19^.

We furthermore assessed the size-specific dose estimate (SSDE) which according to Christner *et al*. reflects the patient dose more independent of size^20^. Analogously to other authors^9,20,21^, the SSDE was calculated by multiplying the CTDI_vol_ with the size-specific conversion factor f_size_ according to the AAPM Report 204^20,22^. The individual f_size_ was obtained by summing the anteroposterior (AP) and lateral (LAT) diameters from transverse CT images at the mid-liver level (size = AP + LAT)^20^.

### Objective image quality

In each patient, the attenuation was measured by a radiologist with 8 years of experience in abdominal imaging (R.F.). In detail, on portal-venous phase images round or oval ROIs were manually placed within the liver (ROI size, 150–300 mm^2^), the pancreas (100–200 mm^2^), the spleen (150–300 mm^2^), the renal cortex (100–200 mm^2^) as well as the abdominal aorta (35–150 mm^2^) and main portal vein (40–80 mm^2^)^15^. For each region, three individual measurements were performed and averaged. Measurements of the liver, the abdominal aorta and main portal vein were conducted at the same level. During measurement, the radiologist carefully avoided focal luminal or parenchymal heterogeneities such as calcifications, thrombotic material, focal lesions, ducts and/or artifacts.

To determine image noise and contrast-to-noise ratio (CNR) of the individual anatomic structure, ROIs with a size of 200–400 mm^2^ were manually placed in the psoas muscles and the adjacent mesenteric fatty tissue. According to Wichmann and colleagues^15^, image noise was defined as the standard deviation (SD) of the ROI in the mesenteric fat (SD_fat_), and the organ-specific CNR was calculated as following:

$${\rm{CNR}}=({{\rm{HU}}}_{{\rm{ROI}}}-{{\rm{HU}}}_{{\rm{psoas}}})/{{\rm{SD}}}_{{\rm{fat}}}$$

As ATVS was enabled, we furthermore calculated figure-of-merit (FOM) values for each organ in both groups in order to provide objective data of differences in CNR independent from the ED^23,24^. The FOM CNR can be calculated using the following formula^15^:

$${\rm{FOM}}\,{\rm{CNR}}={{\rm{CNR}}}^{2}/{\rm{ED}}{\rm{.}}$$

### Evaluation of subjective image quality and motion artifacts

All portal-venous abdominal CT images were read in consensus by two radiologists with 8 (R.F.) and 14 (C.G.T.) years of experience in abdominal imaging. Images were randomly analyzed with freely adjustable window settings. The readers were blinded to the individual patient data and CT reports.

Subjective image quality was evaluated by using a five-point scale according to Guimaraes *et al*.^25^: 1, excellent image quality; 2, good image quality; 3, fair but comprised image quality; 4, poor image quality; 5, non diagnostic, severe distortion.

Similar to others^12^, motion artifacts were categorized as ‘none’, ‘minor’ or ‘major’. Minor motion artifacts were defined as ‘moderately reduced image quality, sufficient diagnostic confidence’, while major motion artifacts were defined as ‘severely reduced image quality, no diagnostic confidence’.

### Influence of size on radiation dose and image quality

For further analysis of radiation dose and image quality, the study population was dichotomized on the basis of the mean size at the mid-liver level (AP + LAT_mean_)^20^. In detail, the calculated AP + LAT_mean_ was 73.6 ± 6.2 cm (72.0–75.2 cm). Accordingly, 33/60 patients were included in group A (AP + LAT < 73.6 cm) and 27/60 patients in group B (AP + LAT > 73.6 cm).

### Statistics

Data analysis was performed using IBM SPSS Statistics for Windows, Version 24.0 (IBM Corp., Armonk, N.Y., USA). A level of significance of α = 0.05 was used throughout the study. Data were initially assessed for normality applying the Kolmogorov-Smirnov test. Continuous variables are provided as mean ± standard deviation (95% confidence interval). Variables that do not follow normal distribution are shown as median (25%; 75% interquartile range).

Variables of the two groups were compared according to the *t* test if data were normally distributed. The Mann-Whitney *U* test was used if data were not normally distributed. The Spearman correlation analysis was applied to investigate the impact of BMI on ED.

## Results

Of the sixty patients included in this study, 28 were female and 32 male. The mean patient age was 56 ± 17 years.

### Patient characteristics

The patient characteristics are summarized in Table 2. The median BMI was 31.5 kg/m^2^ (30.8 kg/m^2^; 33.5 kg/m^2^) in group A and 36.8 kg/m^2^ (33.2 kg/m^2^; 40.2 kg/m^2^) in group B (p < 0.001). Difference in patient’s height between groups did not reach statistical significance (p = 0.051).

**Table 2:** Patient characteristics.

	Group A AP + LAT < 73.6 cm; n = 33	Group B AP + LAT > 73.6 cm; n = 27	P-value
Age, years	57 ± 18 (51–64)	54 ± 17 (47–61)	0.467
BMI, kg/m^2^*	31.5 (30.8; 33.5)	36.8 (33.2; 40.2)	**<0.001**
Weight, kg	91.6 ± 13.4 (86.8–96.3)	112.0 ± 13.2 (106.8–117.2)	**<0.001**
Height, cm	167.7 ± 10.6 (164.0–171.5)	173.1 ± 10.2 (169.1–177.1)	0.051

Normally distributed data are shown as mean ± SD (95% confidence interval). *Data without normal distribution are provided as median (25%; 75% interquartile range). Abbreviations: AP, anteroposterior; LAT, lateral; BMI, body mass index; SD, standard deviation.

Patient characteristics. Normally distributed data are shown as mean ± SD (95% confidence interval). *Data without normal distribution are provided as median (25%; 75% interquartile range). Abbreviations: AP, anteroposterior; LAT, lateral; BMI, body mass index; SD, standard deviation.

### Radiation dose

**Figure 1:**
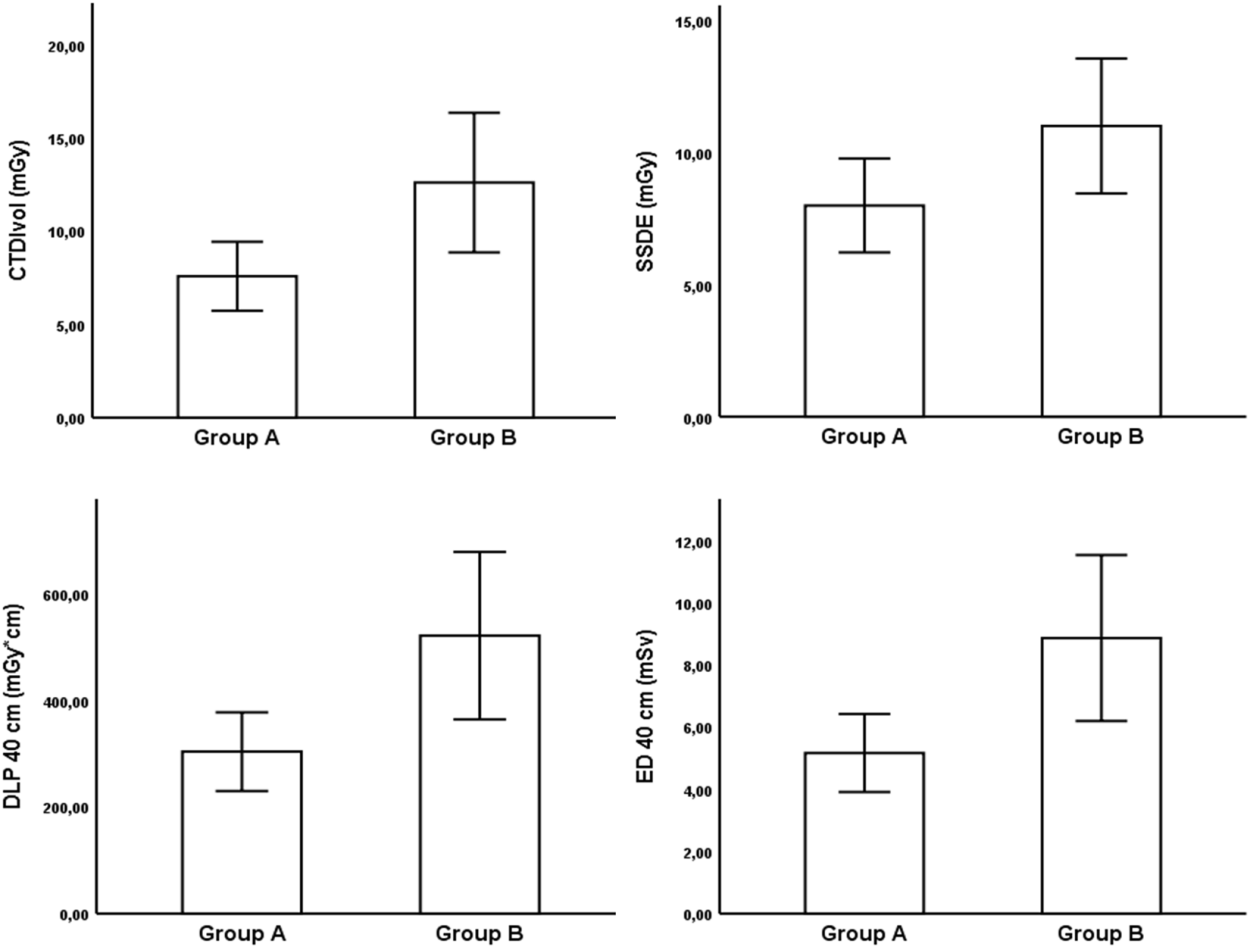
Comparison of CTDI_vol_, SSDE, DLP_40cm_ and ED_40cm_ between group A (AP + LAT < 73.6 cm) and B (AP + LAT > 73.6 cm). Data are shown as mean ± SD. All variables were significantly higher in group B (p < 0.001).

Table 3 and Fig. 1 provide data on radiation dose for both groups. In group A, median selected tube voltage was 100 kV (90 kV; 100 kV) and mean selected tube current time product 213.6 ± 39.4 mAs (199.7–227.6 mAs), while group B was characterized by a median selected tube voltage of 100 kV (100 kV; 120 kV) and a mean selected tube current time product of 259.0 ± 45.4 mAs (241.0–277.0 mAs) (p < 0.001). Minimum and maximum values of selected tube voltage were 80 kV (n = 1, group A) and 130 kV (n = 3, group B).

**Table 3:** Scan length, tube parameters and radiation dose.

	Group A AP + LAT < 73.6 cm; n = 33	Group B AP + LAT > 73.6 cm; n = 27	P-value
Scan length, cm	47.7 ± 3.7 (46.4–49.0)	48.6 ± 4.6 (46.8–50.4)	0.422
Selected tube voltage, kV*	100 (90; 100)	100 (100; 120)	<**0.001**
Selected tube current, mAs	213.6 ± 39.4 (199.7–227.6)	259.0 ± 45.4 (241.0–277.0)	<**0.001**
CTDI_vol_, mGy	7.58 ± 1.84 (6.92–8.23)	12.60 ± 3.74 (11.12–14.07)	<**0.001**
SSDE, mGy	7.99 ± 1.78 (7.36–8.62)	10.99 ± 2.55 (9.99–12.00)	<**0.001**
DLP, mGy * cm	363.7 ± 101.5 (327.8–399.7)	630.6 ± 182.4 (558.5–702.8)	<**0.001**
DLP 40 cm^#^, mGy * cm	303.8 ± 73.9 (277.6–329.0)	521.2 ± 157.1 (459.1–583.4)	<**0.001**
ED 40 cm^#^, mSv	5.16 ± 1.26 (4.72–5.61)	8.86 ± 2.67 (7.80–9.92)	<**0.001**

Normally distributed data are shown as mean ± SD (95% confidence interval). *Data without normal distribution are provided as median (25%; 75% interquartile range). ^**#**^Normalization for a typical abdominal CT scan length of 40 cm. Abbreviations: AP, anteroposterior; LAT, lateral; CTDI_vol_, volumetric computed tomography dose index; SSDE, size specific dose estimate; DLP, dose length product; ED, effective dose; SD, standard deviation; CT, computed tomography.

Scan length, tube parameters and radiation dose. Normally distributed data are shown as mean ± SD (95% confidence interval). *Data without normal distribution are provided as median (25%; 75% interquartile range). #Normalization for a typical abdominal CT scan length of 40 cm. Abbreviations: AP, anteroposterior; LAT, lateral; CTDI_vol_, volumetric computed tomography dose index; SSDE, size specific dose estimate; DLP, dose length product; ED, effective dose; SD, standard deviation; CT, computed tomography.

Mean values of CTDI_vol_, SSDE and DLP were 7.58 ± 1.84 mGy (6.92–8.23 mGy), 7.99 ± 1.78 mGy (7.36–8.62 mGy) and 363.7 ± 101.5 mGy * cm (327.8–399.7 mGy * cm) for group A, and 12.60 ± 3.74 mGy (11.12–14.07 mGy), 10.99 ± 2.55 mGy (9.99–12.00 mGy) and 630.6 ± 182.4 mGy * cm (558.5–702.8 mGy * cm) for group B (p < 0.001).

Using a 40 cm normalization, mean values for DLP and ED were 303.8 ± 73.9 mGy * cm (277.6–329.0 mGy * cm) and 5.16 ± 1.26 mSv (4.72–5.61 mSv) in group A in comparison to 521.2 ± 157.1 mGy * cm (459.1–583.4 mGy * cm) and 8.86 ± 2.67 mSv (7.80–9.92 mSv) in group B (p < 0.001).

Regarding the total study population (n = 60), we observed a significant positive correlation between BMI and ED (r_s_ = 0.583, p < 0.001).

### Objective image quality

**Figure 2:**
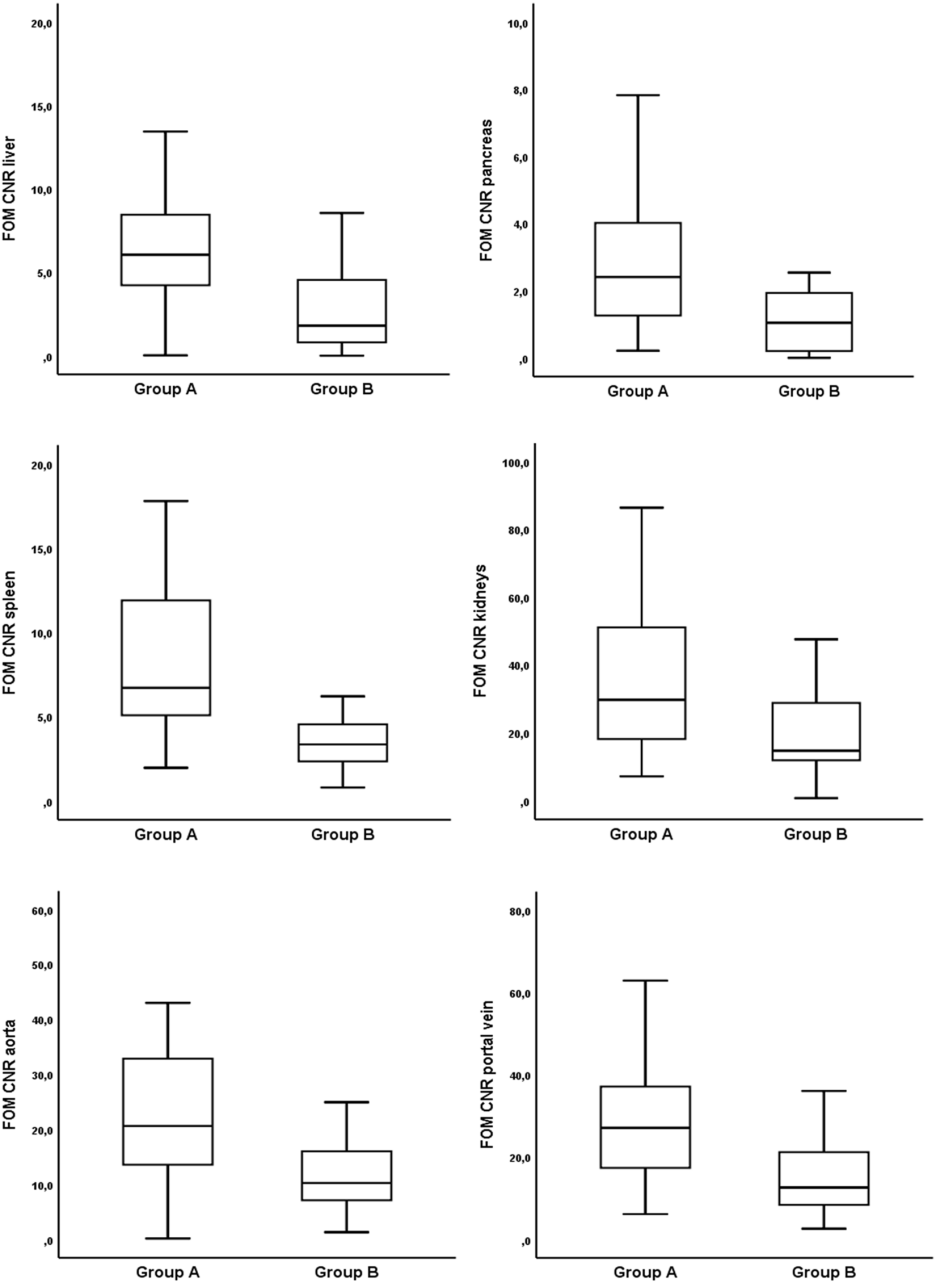
Figure of merit contrast-to-noise ratios (FOM CNRs) for various abdominal organs in group A (AP + LAT < 73.6 cm) and B (AP + LAT > 73.6 cm). Data are shown as median (25%; 75% interquartile range). All calculated values were significantly higher in group A (p < 0.001).

Results from objective analysis of image quality are illustrated in Table 4 and Fig. 2. Significant differences of mean values were observed regarding CNR of the liver (group A: 5.58 ± 1.95 (4.89–6.27); group B: 3.93 ± 2.17 (3.07–4.79); p = 0.003) and spleen (group A: 6.43 ± 1.8 (5.79–7.07); group B: 5.44 ± 1.55 (4.83–6.05); p = 0.027). All other pairwise comparisons of organ-specific CNR (pancreas, kidneys, aorta, portal vein) did not reach statistical significance (p ≥ 0.120).

**Table 4:** Objective image quality results.

	Group A AP + LAT < 73.6 cm; n = 33	Group B AP + LAT > 73.6 cm; n = 27	P-value
**CNR**			
Liver	5.58 ± 1.95 (4.89–6.27)	3.93 ± 2.17 (3.07–4.79)	**0.003**
Pancreas	3.62 ± 1.83 (2.97–4.27)	2.90 ± 1.85 (2.17–3.63)	0.133
Spleen	6.43 ± 1.8 (5.79–7.07)	5.44 ± 1.55 (4.83–6.05)	**0.027**
Kidneys	12.95 ± 4.0 (11.53–14.37)	11.82 ± 3.94 (10.26–13.38)	0.280
Aorta	10.58 ± 3.56 (9.32–11.84)	9.30 ± 2.46 (8.33–10.28)	0.120
Portal vein	11.75 ± 3.24 (10.6–12.9)	10.62 ± 3.15 (9.37–11.86)	0.180
FOM CNR*			
Liver	6.1 (4.0; 8.9)	1.8 (0.8; 5.0)	<**0.001**
Pancreas	2.4 (1.3; 4.2)	1.0 (0.2; 2.0)	<**0.001**
Spleen	6.7 (5.0; 11.9)	3.4 (2.3; 4.6)	<**0.001**
Kidneys	29.7 (17.8; 54.0)	14.7 (11.8; 30.7)	<**0.001**
Aorta	20.6 (13.5; 34.1)	10.3 (7.0; 17.2)	<**0.001**
Portal vein	27.1 (17.0; 37.3)	12.6 (8.4; 21.7)	<**0.001**

Normally distributed data are shown as mean ± SD (95% confidence interval). *Data without normal distribution are provided as median (25%; 75% interquartile range). Abbreviations: AP, anteroposterior; LAT; lateral; CNR, contrast-to-noise ratio; FOM, figure of merit; SD, standard deviation.

Objective image quality results. Normally distributed data are shown as mean ± SD (95% confidence interval). *Data without normal distribution are provided as median (25%; 75% interquartile range). Abbreviations: AP, anteroposterior; LAT; lateral; CNR, contrast-to-noise ratio; FOM, figure of merit; SD, standard deviation.

Regarding dose-independent FOM CNR, the values in group A were significantly higher for all measured organs when compared to group B (p < 0.001). The greatest difference was calculated for the liver with a FOM CNR increment factor of 3.4.

### Subjective image quality

**Figure 3:**
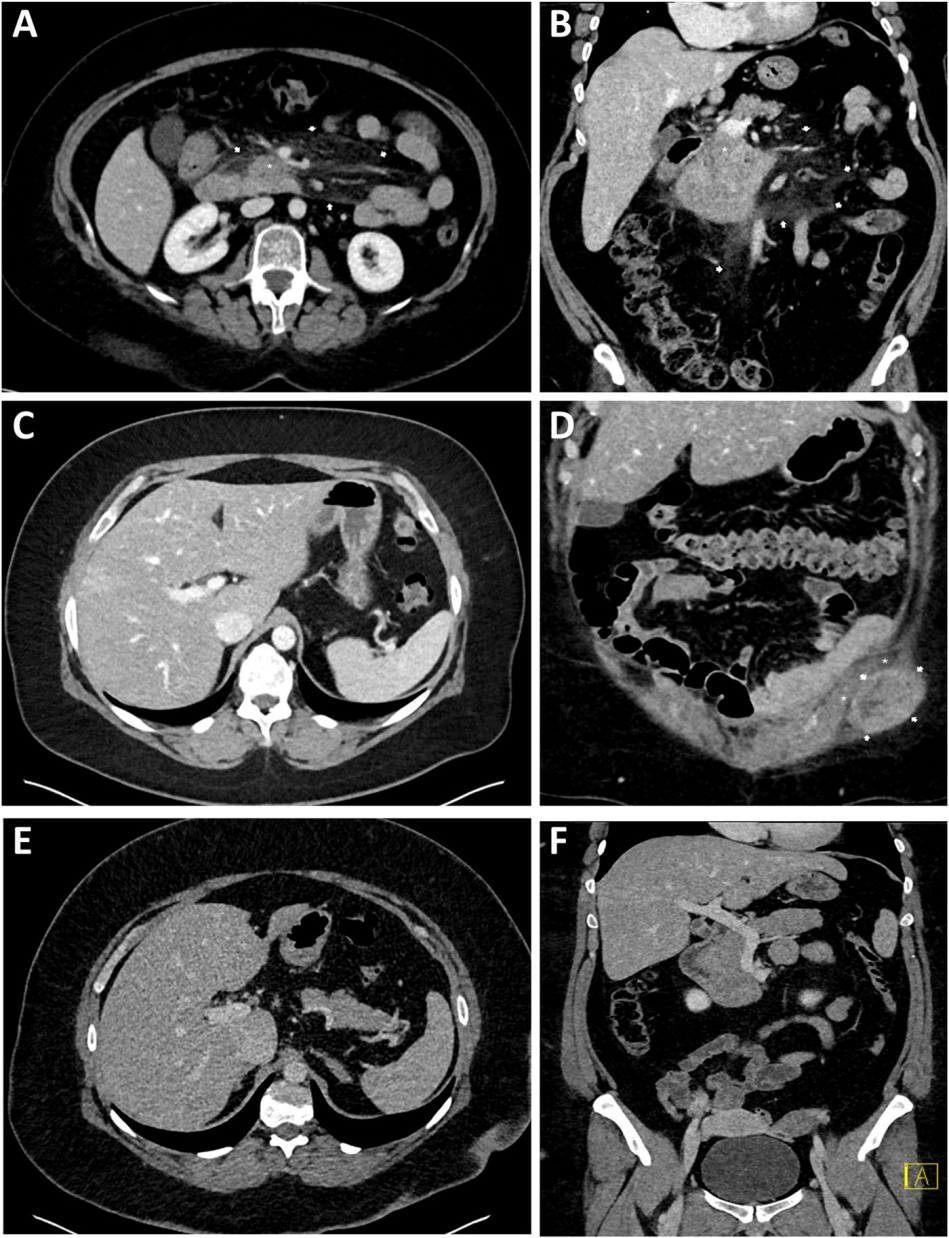
Figures **(A**,**B)** illustrate a high-pitch portal-venous abdominal CT of a 59-year old female patient with acute pain in the upper abdomen (BMI 31 kg/m^2^, AP + LAT 70 cm; CTDI_vol_ 6.38 mGy, SSDE 6.64 mGy, DLP_40cm_ 222 mGy * cm, ED_40cm_ 3.77 mSv; (**A)**, transversal; (**B)**, coronal), showing both slight swelling of the pancreatic head (*) and diffuse stranding of the adjacent mesenteric fat (arrows). Marked elevation of serum lipase confirmed the suspected diagnosis of acute pancreatitis. Image quality was rated ‚excellent‘. (**C**,**D**) show a high-pitch portal-venous abdominal CT of a 41-year old female patient with history of leiomyosarcoma and acute persistent pain in the left lower abdomen (BMI 41 kg/m^2^, AP + LAT 81 cm; CTDI_vol_ 14.13 mGy, SSDE 12.15 mGy, DLP_40cm_ 587 mGy * cm, ED_40cm_ 10.00 mSv; (**C)**, transversal; (**D)**, coronal). In this CT examination, an extraabdominal mass adjacent to the abdominal muscles on the left was diagnosed, in the sense of an abdominal wall metastasis (arrows in **D**). The contrast-enhancing tumor had a well-defined morphology, semi-liquid components, and yielded diffuse stranding of the adjacent fatty tissue. The abdominal muscles (* in **D**) were slightly translocated due to the mass effect, but sharply definable without signs of tumor infiltration. The image quality was rated ‚good‘ in this patient. Figures **(E**,**F)** show a high-pitch portal-venous abdominal CT of a 35-year old male patient with acute pain in the right upper abdomen (BMI 47 kg/m^2^, AP + LAT 86 cm; CTDI_vol_ 17.34 mGy, SSDE 13.53 mGy, DLP_40cm_ 720 mGy * cm, ED_40cm_ 12.24 mSv) (**E)**, transversal; (**F)**, coronal). The CT scan revealed no pathology, particularly no signs of cholecystitis. Overall image quality was rated ‘good’.

Table 5 and Fig. 3 provide results of subjective image quality evaluation. According to two readers, the median rating of overall image quality was 2 (good) in both groups; however, difference of ratings between groups reached significance towards a higher image quality in group A (p = 0.035). In detail, examinations in group A were exclusively rated as 1 (n = 13/33) or 2 (n = 20/33), whereas ratings in group B were 1 in 6/27, 2 in 16/27, and 3 in 5/27 examinations. Of the five patients in whom the image quality was rated 3, the mean BMI was 44 kg/m^2^. For both groups, no minor or major motion artifacts were documented.

**Table 5:** Subjective image quality evaluation.

	Group A AP + LAT < 73.6 cm; n = 33	Group B AP + LAT > 73.6 cm; n = 27
1 (excellent)	13	6
2 (good)	20	16
3 (fair)	0	5
4 (poor)	0	0
5 (non diagnostic)	0	0

Abbreviations: AP, anteroposterior; LAT; lateral.

Subjective image quality evaluation. Abbreviations: AP, anteroposterior; LAT; lateral.

## Discussion

In the present study we investigated radiation dose and image quality of portal-venous high-pitch emergency CT of the abdomen in obese patients on a third-generation DSCT scanner. We believe, that our findings add relevant information to the field of emergency abdominal CT in overweight individuals, as comparable data are missing. Our results suggest, that the large tube generator capacities of third-generation DSCT in combination with the high-pitch (Flash) mode and iterative reconstruction enable abdominal CT examinations of overweight patients, yielding both good image quality and a reasonable radiation dose.

It has been demonstrated, that third-generation DSCT provides a dose reduction of at least 30% in portal-venous abdominal CT using a pitch factor of 0.6 when compared to second-generation DSCT^15^. Furthermore, as the high-pitch dual-source mode (in combination with a reduced rotation time) of third-generation DSCT allows for a shorter CT acquisition time^10^, this technique probably further optimizes radiation dose efficacy in comparison to a standard-pitch protocol^8–10,26,27^. Likewise, in the present high-pitch study the ED of the group with a lower BMI (median BMI 31.5 kg/m^2^) was yet below the value measured by Wichmann and colleagues who applied similar tube settings but a standard-pitch protocol on the same third-generation DSCT scanner^15^, even though the BMI was lower in their study (this study: mean ED 5.2 mSv; Wichmann *et al*.: mean BMI 27.6 kg/m^2^, mean ED 6.2 mSv). However, as ATCM and ATVS were enabled in our study, the scanner output was automatically adjusted depending on the imaged object and size^20^. Accordingly, we found a positive correlation between BMI and radiation dose. Furthermore, the mean values of CTDI_vol_ and DLP were significantly increased in the group with a higher BMI (median BMI 36.8 kg/m^2^) when compared to the group with a lower BMI, resulting in a mean ED of 8.9 mSv in the former group.

For DLP-based calculation of ED, we applied the combined conversion factor for abdominal and pelvic CT according to the ICRP 60 publication (*k* = 0.017 mSv/mGy * cm)^18,19^ for reasons of comparability with regard to relevant published studies^14,15^. Application of the more recent ICRP 103 publication might have yielded a further 7% reduction of ED in both groups according to Christner *et al*.^19^; however, this adjustment actually would not have altered the overall conclusion of the present study. Furthermore, the calculated ED in our obesity study (BMI ≥ 30 kg/m^2^) is possibly overestimated at least in the group with a higher BMI and AP + LAT diameter (> 73.6 cm), respectively, as their organs are more strongly shielded against X-radiation due to the distinct fatty tissue when compared to slimmer individuals^28^. For a more precise estimation of the ED conversion factor in patients with different BMIs we therefore recommend the application of specific obesity phantoms^28^. Nevertheless, mean values of other dose descriptors assessed in this study (CTDI_vol_ and DLP), which are more objective than the ED, were still below the diagnostic reference levels provided by the respective federal office for radiation protection (CTDI_vol_ 15 mGy, DLP 700 mGy * cm)^29^ even in the group with a higher BMI.

The SSDE, representing another dose descriptor which can be derived from the CTDI_vol_^20^, considers the individual patient size and thus reflects more precisely the mean absorbed patient dose. To note, the calculated mean of the sum of AP and LAT diameters defining the cut-off for the two groups (lower BMI and higher BMI) was 73.6 cm in the present study. According to Table 1 of AAPM Report 204, this dimension almost exactly corresponds to a SSDE conversion factor of 1 and hence to the CTDI_vol_ obtained for a standard 32 cm diameter phantom^22^. We assessed a significant increase of SSDE in the group with a higher BMI and diameter, respectively – although less distinct when compared to the increase of CTDI_vol_. These findings contrast with Christner *et al*.^20^ who suggested that the SSDE is more or less independent from patient size. However, Li *et al*.^21^, who created whole-body computational phantoms from clinical images of normal- and overweight patients, showed that obesity has a significant effect on dose coefficients, that cannot be predicted using only the body diameter, and that SSDE overestimates organ dose for obese patients.

Regarding dose optimization – besides iterative reconstruction^8,9,26^ – reduction of tube voltage represents a well-established technique as it increases the intravascular contrast, which is of particular interest in CT angiography. With third-generation DSCT, for example, Meinel *et al*. demonstrated that lowering the tube voltage from 120 kV to 70–80 kV in coronary CT angiography reduces overall radiation dose by 49–68% while maintaining CNR even in obese patients^7^. In the present study, attenuation-based ATVS was enabled (in addition to ATCM) throughout the study period as mentioned above. ATVS optimizes – based on the attenuation along the z-axis according to the topogram – dose efficiency dependent on patient-specific mAs curves for all kV levels^17^. In third-generation DSCT, it has been shown that ATVS mostly selects lower kV values in body CT angiography of normal-weight individuals when compared to second-generation DSCT (90 versus 100 kV), consequently yielding further dose reduction^17^. In this obesity study, the median tube voltage selected by ATVS was 100 kV in both groups, which also represents a commonly applied value for non-weight selected portal-venous DSCT studies^15^. However, differences between groups in the present study reached statistical significance towards increased automatically selected values of both tube voltage (maximum 130 kV) and tube current time product in the group with a higher BMI.

Regarding objective image quality, we calculated CNRs and FOM CNRs of various organs and vessels. CNRs of the two parenchymal upper abdominal organs liver and spleen were significantly higher in the group with a lower BMI when compared to the group with a higher BMI. This was possibly caused by the relatively superficial anatomical location of these two organs resulting in a lower image noise in the former group. Furthermore, the dose-independent FOM CNR^24,25^ actually revealed a distinct difference of objective image quality between groups for each assessed organ, with significantly higher FOM CNRs for the group with a lower BMI. These FOM CNRs also exceeded the calculations by Wichmann *et al*. who applied a standard-pitch protocol in slimmer patients on the same third-generation DSCT scanner^15^. Nevertheless, this finding had limited clinical relevance in the present study, as the image quality was high in the majority of examinations even in the group with a higher BMI despite comparably lower FOM CNRs. We indeed noted increased image noise in patients with the highest BMIs (maximum BMI in this study: 54 kg/m^2^), but overall image quality remained diagnostic in these examinations. To note, in this most obese patient ATCM yielded a maximum combined tube current at the umbilical region of 2488 mA according to the DICOM header. Thus, we state that the tube current limit of both X-ray tubes (2 × 1300 mA) was not reached in this study. Altogether, the applied high-pitch DSCT protocol was robust to confirm (or rule out) therapeutically relevant pathologies of parenchymal organs and the gastrointestinal tract in both groups.

High-pitch DSCT protocols with pitch factors of at least 1.9^12^ allow for a reduction of motion artifacts which is of particular interest in chest CT. In the present abdominal obesity study with a preset pitch of 1.55, no minor or major motion artifacts were documented. The applied pitch, which is still clearly higher than in standard-pitch protocols^15^, therefore seems to be sufficiently high for abdominal CT. In this context, with previous CT scanner models the pitch had to be decreased in order to accumulate radiation dose and increase image quality in CT examinations of obese patients, if the tube had reached its maximum power limit^3^. In this study, it was not necessary to further decrease the pitch despite the large patient size, as the tube generator of the utilized third-generation DSCT scanner has larger capacities^7^.

The results of the present study should be interpreted in the context of the study design and its limitations. First, CT data were only collected from one specific vendor (Siemens Healthineers), and we neither compared high- with standard-pitch CT acquisition protocols nor high- with standard-BMI patients. Thus, further studies are recommended in order to increase generalizability. Second, we did not apply a dedicated low-kV protocol due to concerns of image quality in this obesity study. However, we believe that a further reduction of tube voltage (e.g., 80 kV) may be reasonable at least in individuals with a BMI lower than 37 kg/m^2^ in order to achieve further dose optimization while maintaining diagnostic confidence. Third, extremely obese patients (weight > 300 kg) were not included, as their positioning is difficult with respect to standard gantry diameters and table weight limits. Future CT development by large manufacturers might cope with the epidemiologic impact of obesity on emergency units. Finally, vascular emergencies were not explicitly evaluated in this portal-venous CT study. However, CNR within large abdominal vessels was high, enabling the diagnosis of therapeutically relevant pathologies.

In conclusion, high-pitch emergency CT of the abdomen in portal-venous phase can be routinely performed in obese patients due to large tube generator capacities of third-generation DSCT. In the present study, radiation dose increased in individuals with a BMI > 36.8 kg/m^2^ as automated dose modulation (ATCM and ATVS) was enabled. This increase did not exceed diagnostic reference levels. Dose-independent FOM CNR was highest for obese patients with a BMI < 36.8 kg/m^2^, while image quality remained sufficient in patients with higher BMIs. The applied high-pitch CT protocol should be adapted according to dedicated dose and image quality optimization methods.

## Data Availability

The datasets generated and/or analyzed during the current study are available from the corresponding author on reasonable request.
